# Donor-funded project's sustainability assessment: a qualitative case study of a results-based financing pilot in Koulikoro region, Mali

**DOI:** 10.1186/s12992-017-0307-8

**Published:** 2017-12-08

**Authors:** Mathieu Seppey, Valéry Ridde, Laurence Touré, Abdourahmane Coulibaly

**Affiliations:** 1 Université de Montréal, École de santé publique (ESPUM), P.O. Box 6128, Succursale Centre-Ville, Montréal, Québec, H3C 3J7 Canada; 2 Institut de Recherche en Santé Publique de l’UdeM (IRSPUM), 7101 Avenue du Parc, Office 3187-03, Montréal, Québec, H3N 1X9 Canada; 3 MISELI (Association Malienne de Recherche et Formation en Anthropologie des Dynamiques Locales), cité el-Farako, BP E5448 Bamako, Mali

**Keywords:** Sustainability, Routinisation, Institutionalisation, Health financing, Results, Mali, Project management

## Abstract

**Background:**

Results-based financing (RBF) is emerging as a new alternative to finance health systems in many African countries. In Mali, a pilot project was conducted to improve demand and supply of health services through financing performance in targeted services. No study has explored the sustainability process of such a project in Africa. This study’s objectives were to understand the project’s sustainability process and to assess its level of sustainability.

**Methods:**

Sustainability was examined through its different determinants, phases, levels and contexts. These were explored using qualitative interviews to discern, via critical events, stakeholders’ ideas regarding the project’s sustainability. Data collection sites were chosen with the participation of different stakeholders, based on a variety of criteria (rural/urban settings, level of participation, RBF participants still present, etc.). Forty-nine stakeholders were then interviewed in six community health centres and two referral health centres (from 11/12/15 to 08/03/16), including health practitioners, administrators, and those involved in implementing and conceptualizing the program (government and NGOs). A theme analysis was done with the software © QDA Miner according to the study’s conceptual framework.

**Results:**

The results of this project show a weak level of sustainability due to many factors. While some gains could be sustained (ex.: investments in long-term resources, high compatibility of values and codes, adapted design to the implementations contexts, etc.) other intended benefits could not (ex.: end of investments, lack of shared cultural artefacts around RBF, loss of different tasks and procedures, need of more ownership of the project by the local stakeholders). A lack of sustainability planning was observed, and few critical events were associated to phases of sustainability.

**Conclusions:**

While this RBF project aimed at increasing health agents’ motivation through different mechanisms (supervision, investments, incentives, etc.), these results raise questions on what types of motivation could be more stable and what could be the place of local stakeholders in the project; all this with the aim of more sustained and efficient results.

## Résumé

**Contexte:**

Le financement basé sur les résultats (FBR) est. un type de financement de plus en plus utilisé dans les systèmes de santé en Afrique. Au Mali, un projet pilote de FBR a été implanté afin d’améliorer l’offre et la qualité des soins, en ciblant des services payés à la performance. Aucune étude n’a encore exploré le processus de pérennisation d’un tel. programme en Afrique. Les objectifs de cette étude étaient de mieux comprendre le processus de pérennisation du projet et d’évaluer son niveau de pérennité.

**Méthodologie:**

La pérennisation a été analysée à travers ses différents déterminants, phases, niveaux et contextes. Quarante-neuf entrevues ont été effectuées dans six centres de santé communautaire et deux centres de santé de référence (de 11/12/15 à 08/03/16) avec des prestataires de soins, gestionnaires et ceux impliqués dans l’implantation et conceptualisation du projet (gouvernement et ONGs). Les sites de collecte de données ont été choisis avec la participation de différentes parties prenantes et sur la base de différents critères (contexte rural/urbain, niveau de participation, participants FBR toujours présents, etc.). Une analyse thématique a été conduite avec le logiciel QDA Miner © en lien avec le cadre conceptuel de l’étude.

**Résultats:**

Les résultats montrent que le niveau de pérennité du projet est. faible pour plusieurs raisons. Lorsque certains gains ont pu être maintenus (ex.: investissements dans des ressources à long-terme, compatibilité des valeurs et codes, design adapté aux contextes d’implantation, etc.), d’autres n’ont pu l’être (ex.: fin des investissements, manque de partage d’artefacts culturels autour du FBR, perte de tâches et procédures FBR, besoin d’appropriation du projet par les parties prenantes). Un manque de planification de la pérennité a été observé, et peu d’événements critiques ont été associés aux phases de la pérennisation.

**Conclusions:**

Si ce projet FBR visait à l’augmentation de la motivation des agents de santé via différents mécanismes (supervision, investissements, primes, etc.), ces résultats amènent à se poser des questions sur quels types de motivation serait plus stables et quelle serait la place des parties prenantes; tout ceci avec le but d’obtenir des résultats plus efficients et pérennes.

## Background

Ways of financing health systems are in constant evolution, depending on needs for efficiency, quality or access to care [1–4]. In Africa, those financing methods are often influenced by international orientations [1–3, 5, 6]. To this day, many international organisations encourage the implementation of results-based financing (RBF) methods to increase health professionals’ productivity and quality of services [1, 2, 4, 6–9]. Supply-centred RBF is the provision of incentives (financial or else) linked to the production and the quality of specific services. Theoretically, this link between incentives and targeted services would allow a better adequacy between supply and demand for care services [7, 10, 11]. There is however no clear consensus around the effects of RBF, depending on the different settings, types of services or RBF designs [12–15]. While research efforts have focused on RBF projects’ effects, very few studies offered a better understanding of the sustainability of such projects, which is an actual gap in the knowledge around RBF [7, 16, 17].

Many questions are currently asked concerning project implementation: are the projects effective, cost-efficient in the long run or do they cease to exist at the end of their budget [18]? The growing study of sustainability starts giving some answers to researchers and project managers but remains very limited and does not offer a consensus around the concept of sustainability [18–20]. Supporting this idea, the concept of sustainability is often distorted in many terminologies: institutionalisation, diffusion, appropriation, consolidation, durability, integration, perpetuation, routinisation, permanency, maintenance, and many more [21]. However, the aspects of temporality and integration to a structure remain preponderant in the conceptualisation of sustainability (as a process and as a status at a point in time) [21–23]. Many models represent sustainability as the main goal of a project [21, 23]. Chambers et al. [24] nonetheless illustrate sustainability as a continued process (in comparison to a goal), fed by a constantly changing context of implementation and a dynamic intervention being modified through time. This model is considered more realistic by better reflecting the organisational reality than a model considering sustainability as a mere final step [24, 25].

A sustainable status can be categorised into five levels: i) null, if no activities from the intervention subsisted within the host organisation, ii) precarious, when some activities subsisted but still are unofficial, dependant from specific actors and do not correspond to the intervention framework, iii) weak, if activities officially continue, but do not systematically show the determinants of a routine and can easily disappear in the short term, iv) moderate, when activities are stable and totally routinized, and v) high when those routinized activities are institutionalised at a higher level of implementation (e.g. national level) [20, 25, 26]. Routinisation represents the first 4 levels of activity integration within a host organisation, all this through four main determinants: organisational memory, values and codes, rules and procedures, and adaptation to contexts [21]. Organisational memory is the ability to maintain the structure of the organisation (social network, archive system, etc.) via stable resources. Values and codes are represented through cultural artefacts (symbols, rituals and languages) that the intervention’s stakeholders share together. Rules and procedures are the framework in which actions are taken and decided for the implementation of the intervention. Finally, adaptation is an intervention’s capacity to correspond to its context in regards to its simplicity, its ability not to perturb the host organisation’s daily activities, etc. Being the fifth degree of sustainability, institutionalisation refers to a systematic level of integration through social norms, standards, legal frameworks, etc. [21]. As a pilot, this project cannot be expected to be evaluated against the goal of institutionalisation. However, it can be assessed in regards to its routinisation process. It is therefore through routines (via its main determinants) that sustainability will be measured; routines being acquired activities by the organisations thanks to the pilot [27].

Very few studies deal with sustainability of developmental projects in Africa [28] and even less concerning RBF projects (as a routinisation process more specifically) [14, 17, 29–31]. Therefore, the objective of this study is to evaluate the degree of sustainability of a RBF pilot project and to better understand its sustainability process.

To respond to these objectives, a case study [32] was made of the RBF pilot project in Mali. Concerning this country, it is administration is perceived as highly decentralised. National, specialised and regional hospitals are public autonomous establishments and represent tertiary health centres at the national level and in the eight regions. Referral health centres (CSREF) refer to the national direction for health (DNS) and are at the health district level. They are managed by the circles’ councils (CC) (circles are the administrative level between regions and villages and correspond more or less to the health districts) and form the secondary line of health care services. First line services are given by the community health centres (CSCOM) which are situated at the health area level and managed by community health associations (ASACO) with communes (or communal councils). They refer to the CSREF [33, 34].

The pilot project went on from February 2012 to December 2013 in the health districts of Dioïla, Fana and Banamba (Koulikoro region) and was implemented by the Ministry of Health and Public Hygiene (MHPH) with the help and funds of the Dutch cooperation (SNV) and the Royal Tropical Institute (KIT). The implementation was divided in 5 phases/payments were a total of 26 CSCOM and 3 CSREF gradually entered the project [35]. This pilot’s objectives were to improve the provision of services and their quality in the region, one of the poorest in the country, and to be the first step of a reform of the national health system [35–37]. A diverse group of actors was involved in this RBF project: health practitioners from CSCOM and CSREF (being the services’ providers), communes and ASACO (as service contractors and buyers), district and regional health managers from the district managers’ team and the regional direction for health (DRS) (as regulators and auditors of the CSCOM and CSREF levels respectively), and different independent international non-governmental organisations (NGO) (playing the role of results counter-auditors) [37]. RBF consisted in granting workers and health organisations performance bonuses linked to the provision of quality services. After buying those services (transferring the bonuses), 40% of the bonuses were redistributed to health care personnel and 60% to the organisations’ managing committee (ASACO or CC) (vice versa in regards to CSREF). The bonuses received by health care personnel could represent more than double the normal revenue [35]. Like other RBF projects, the pilot on Mali also included components such as the reinforcement of the organisations’ monitoring and evaluation system, a greater participation and autonomy of local authorities, committees and health agents in the organisations’ management, as well as the clarification of different procedures, roles and activities [38]. At the end of the project, a significant increase of service utilisation was seen, particularly with the healthy children’s monitoring (SES) or the third post-natal consultations (CPON). This pilot project is part of a larger reform of the health system, aiming to establish payments after services’ provision and evaluation [35].

## Methods

### Conceptual framework

**Fig. 1:**
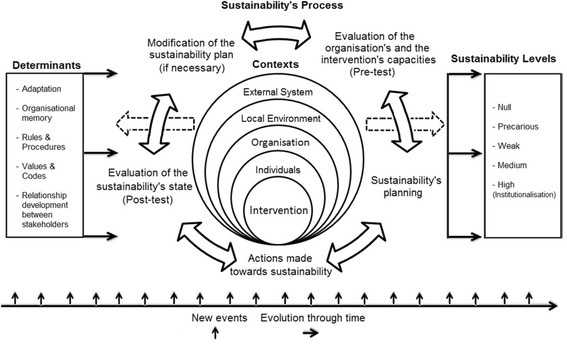
Sustainability framework. (Adapted from Johnson et al. [22], Chambers et al. [24], Pluye, Potvin and Denis [21] and Moullin et al. [23])

The conceptual framework used for this study comes from the integration of the propositions of Johnson et al. (2004), Chambers et al. (2013), Pluye, Potvin and Denis (2004) et Moullin et al. (2015) [21–24] [see Fig. 1]. Our framework proposes a representation of sustainability as a processual concept including: i) its determinants, ii) the process of sustainability, iii) the contexts within which sustainability takes place, iv) critical events influencing sustainability, and v) degrees of sustainability resulting from the previous elements.

First, according to the literature, five determinants are to be considered in regards to the capacity of an intervention to be sustainable: i) the adaptation to the different contexts of implementation, ii) the creation of organisational memory, iii) the match between values and codes of the intervention and the ones of the host organisation, iv) the establishment of rules and procedures, and v) the relationship between the project stakeholders [22, 24, 25]. The development of relationships between the project’s stakeholders takes into account many elements: creation/reinforcement of the stakeholders’ networks, communication, project appropriation by the stakeholders, their specific motivation/benefits, and leadership.

Second, due to the evolving context of the intervention, the sustainability process must be continued throughout its different phases: i) the evaluation of the integration’s capacities of both the host organisation and the intervention, ii) the planning of sustainability, iii) the execution of actions towards sustainability, iv) the evaluation of the current degree of sustainability, and v) the modifications of planning/actions towards sustainability [22].

Third, sustainability must be linked to its contexts that can be categorised into five different implementation contexts, going from the most intervention-centred to the most general: i) the intervention itself, ii) the individuals working directly in the intervention, iii) the host organisation englobing the intervention, iv) the local environment and its different actors, and v) the external system of values (politics, economics, laws, morality). Those implementation contexts integrate sustainability determinants and its process, as well as the degree of sustainability.

Fourth, critical events are also integrated into this framework since they can directly influence implementation contexts or the different determinants of sustainability. Critical events are normally shared between actors and therefore represent turning points in the history of a project. Concerning sustainability, different events are of particular interest; events related to the project’s standardisation (its institutionalisation), the stabilisation of its resources, risks taken for the project, the integration of its rules and procedures within the organisation, the motivation of actors to get involved in the project, its adaptation to the context of implementation, the correspondence of objectives between the organisation and the project, transparent communication and the sharing of the project’s cultural artefacts. Finally, degrees of sustainability are divided into five which were described previously.

### Data collection

The pilot project took place in 29 health centres in the health districts of Dioïla, Fana and Banamba. Collection sites were selected based on their ability to provide maximum variation sampling. This was performed with the help of key informants working in two CSREF and according to different criteria: physical access, diversification of sites (urban vs. rural; high vs low performance through service indicators; deviant cases), availability of data (low personnel rotation), and security. For security reasons, no site was selected in the Banamba district, leaving the sample to be comprised of the two CSREF of Dioïla and Fana and three CSCOM per district (6 CSCOM in total).

For every site, between 4 and 5 participants were interviewed: management, health care practitioners and ASACO or CC members. The CSCOM’s technical directors (DTC) (being the only member of management) and CSREF’s member of the administration served as key informants in the recruitment of health care practitioners and ASACO or CC members. Participants’ selection was made upon their knowledge and experience regarding the pilot project. Other participants were subsequently recruited to deepen the understanding of the role of other organisations involved in the implementation of the project: the DNS, the DRS of Koulikoro, the SNV, the KIT and the communes. In total, 49 semi-structured interviews were conducted lasting between 30 min and 2 h [see Table 1]. Interviews were individual and within the work place or the health centre. While the audio was recorded, notes were also taken during the interviews for better data analysis. An interpreter was also trained by a senior researcher to facilitate interviews in Bambara (AC). An interview guide was constructed by the research team (MS, VR, LT, AC) and validated through a pre-test with three personnel members of a CSCOM. Minor modifications were made and pre-test data was also used for the analysis. This guide comprised five sections according to the sustainability’s determinants. Open ended questions were used to collect data on sustainability’s determinants, phases, contexts and critical events (elements based on the conceptual framework described above).

**Table 1:** Participants sampling

		Districts	
		Fana	Dioïla
Health areas (CSCOM)	Management	3	4
	Health care personnel	8	8
	ASACO	3^a^	4^a^
Health districts (CSREF)	Management	1	1
	Health care personnel	3	3
	Dioïla CC (including Fana’s CSREF)	2	
Communes	Mayors, commissioners, general secretaries	2	2
Directions	DNS & DRS members	2	
Implementation	SNV & KIT members	3	
Total		49	

^a^One of the interviews was conducted with two participants (both ASACO’s president and treasurer were interviewed)

Both audio recordings and interview notes were transcribed *verbatim*. Audio recordings were also re-played and listened to and the *verbatim* re-read (MS) (exception of the Bambara interviews) to ensure their validity and integrity, as well as to perform an iterative analysis of the data. The © QDA Miner software was utilised to manage data and help for the thematic analysis. A code tree was created (MS) and reviewed (VR) according to the conceptual framework of the study [32]. This tree was refined with the addition of new themes emerging from the iterative analysis. Preliminary results were presented to different actors linked to RBF during a workshop in Bamako [39]. Comments helped to identify other actors and to validate some data and analysis.

A documentary research was also done to triangulate data from the interviews and to better understand the functioning of the project. Few documents were available due to confidentiality and administrative constraints.

Precautions were also taken during the study to follow different rigorous criteria such as the credibility, authenticity or integrity of the results. To ensure transferability, details relating to the context, process, intervention, etc. were also offered. This study was approved by the ethical committee for health research of the Université de Montréal (15–145-CERES-D) and by the DNS in Mali (#3269). Participants’ consent was informed, free and continued. Results stayed anonymous and confidential at every step of the study.

## Results

After the iterative analysis of data, results showed that the RBF pilot project in Mali had weak degree of sustainability, which will be explained through the following empirical data. Table 2 presents a synthesis of the different critical events, according to the implementation contexts, determinants and processes.

**Table 2:** Results synthesis

Implementation contexts	Determinants	Processes
Intervention	*Organisational memory:* - Sufficient amount of resources for the project’s implementation - Instable resources (foreign agencies) *Adaptation:* - Design of the intervention adapted to the different contexts - Simplicity of the design *Dev. of relationships:* - Important amount of stakeholders united for the intervention	*Evaluation of the integration capacity:* - Over reliance on foreign investments/prices - Design mapping process by KIT *Sustainability planning:* - Project understood in a determined period of time and experimental with no sustainability attached
Individuals	*Organisational memory:* - Increased expertise (trainings and feedbacks from supervisions) - Improved work conditions (long and short terms investments in infrastructures and materials) *Values/Codes:* - Few cultural artefacts shared between actors - Problem of language between sub-groups of stakeholders *Rules/Procedures:* - Clarity of the tasks (new routines) and roles - Regular supervisions - Low use of the implementation tools by health workers (the supervisor takes responsibilities) *Adaptation:* - Correspond individuals’ needs (ex.: bonuses and better work conditions) - Low critical perspective towards the project (absence of negative effects) *Dev. of relationships:* - High motivation of health workers (bonuses)	*Execution of action towards sustainability:* - High rotation of personnel leading to few actions towards sustainability - Cessation (or diminution) of the bonuses, supervisions, trainings, investments
Organisation	*Organisational memory:* - Punctual integration of new resources (financial, infrastructures, materials, staff, etc.) - Few risks taken for the project (ex.: investments and demand of credits) *Values/Codes:* - New rituals put in place (ex.: meetings, supervisions) - Correspondence of RBF’s goals and the organisation’s (ex.: improve performance of health centres) *Rules/Procedures:* - Reinforcement of old routines and instituting new ones (ex.: PMA) *Adaptation:* - Similarity of new and old routines - Clear system of monitoring and evaluation - Few adaptations of the project by the organisations (ex.: new registries, awareness campaigns) - Goals setting according to the organisation *Dev. of relationships:* - Unequal benefits to the stakeholders (ex.: bonuses for health workers and moral recognition for communes) - Intra-organisation cooperation during the project	*Evaluation of the integration capacity:* - Evaluation of performance only based over 3 months prior the project *Execution of action towards sustainability:* - Sustained routines (better reception, punctuality, use of registries) - Few actions taken to sustain routines (ex.: talks in favour to maintain results rather than routines)
Local environment	*Organisational memory:* - Local investments to prepare RBF (ex.: materials, new staff, trainings) *Values/Codes:* - Absence of shared cultural artefacts *Dev. of relationships:* - Temporary reunion of stakeholders (ex.: communes, women associations, health workers) - Competition between organisations - Few cooperation between organisations	*Evaluation of the integration capacity:* - Local particularities not taken into account during first evaluation *Sustainability planning:* - No planning to sustain the new relationships
External system	*Organisational memory:* - Trainings at national/regional levels - Resources controlled by foreign agencies *Values/Codes:* - Correspondence of RBF’s goals and the health system’s (ex.: improve health indicators) *Adaptation:* - From Rwandan RBF to “*à la malienne”* (decentralised version of RBF) - Embedded in the health system apart from the budget management *Dev. of relationships:* - Lack of involvement of local authorities (ex.: communes, governmental agencies)	*Evaluation of the integration capacity:* - Lack of local leadership in making a baseline study for the project (after USAID withdrew from the project because of the *coup d’État*) *Sustainability planning:* - Dependence on international support to maintain the gains of the project *Evaluation of sustainability and modifications:* - Sustainability is only considered through a scaling up operation

### Sustainability determinants

#### Organisational memory

“*Who writes well has a good pen!*” (DTC, CSCOM Test).^1^

Many types of investments improved the quality and quantity of services: material resources (construction of maternity wards, medical furniture, motorbike repairs, access to water and electricity, etc.), human resources (trainings, personnel recruitments, etc.), informational resources (population awareness-raising sessions concerning RBF, inter-CSCOM meetings, etc.) and financial resources (private loans, contributions). Those investments came from RBF purchases but also, to a lesser extent, from ASACOs’ proper funds to ensure greater subsequent purchases by the RBF project. For example, many obstetrical nurses were recruited before the first purchase of services to ensure quality deliveries. Many vehicles were also repaired to guarantee the “*Advanced strategies*” (AS), which are mobile clinics reaching the most remote communities with services such as family planning (FP), vaccination or pre/post-natal consultations (CPN and CPON). Many investments in buildings (hangars, renovations, houses, etc.) stabilised already acquired resources: “*…the panels* [solar]*, we know that it is a sure investment.*” (DTC, CSCOM 1). In the context of limited resources, it is necessary to maximise investments in time; “*…equipment, when we have it, it lasts… unless it is broken.*” (ASACO/president, CSCOM 4).

Investments responded to the different actors’ needs: work materials, training for health care practitioners, improved quality/quantity of services, awareness-raising campaigns promoting health centres services, etc. An increased attendance of the health centres was perceived by the different RBF stakeholders, which therefore stabilised the centres’ revenues and even augmented them: “*Results that we had with RBF, it is always* [thanks to] *the awareness-raising campaigns… in 2014, 2015* [after RBF]*, with consultations, there was even an increase, which is through awareness-raising campaigns.*” (Pharmacy manager, CSCOM 6).

In preparation for RBF, workshops were given to different stakeholders to perfect their knowledge in regards to their respective roles within the project. The level of expertise was however low since “*…everyone was new with the concepts.*” (Member 2, KIT). Some components of the project had to be explained again through additional workshops to increase comprehension: “*I* [a hypothetic manager] *have the pre-financing budget, and then on top of that, also you get your primes de performance, but that's just after a quarter. So there in budgeting terms, there was quite confusion at the beginning… and that makes financial planning more complicated.*” (Member 2, KIT).

Few organisational risks were taken during the implementation of RBF. Despite being perceived as risky (large amounts of money, uncertainty regarding the purchase of the services), some investments were made with the aim of stabilising and increasing the resources of the centres (construction of laboratories for analysis, awareness-raising campaigns, etc.).

#### Values & Codes

“*When you called me and said RBF, there… directly I saw that… directly: good work and good results!*” (Mayor, CSCOM 4).

According to the majority of participants, the objectives of health care centres and those of RBF were “*together*” (corresponding). Health care centres’ objectives consisted mainly to provide the minimum package of activities (PMA) and to be responsible for the population’s health, “*…approaching health structures to the population…*” (Mayor, CSCOM 4). Through RBF, the objective of quality was added while focusing on health prevention/promotion. Throughout the purchase of services, the personnel’s motivation also seemed to be an objective.

The sharing of cultural artefacts concerning RBF was linked to the perceived project’s effects: investments, service improvements (punctuality, 24 h services, well done tasks, etc.) and the “*buru futini* 2 [small bread]/*prime*” (financial motivation). Results evaluations were integrated in a certain language: “*If you put such behaviour and that the “RBF mogow”* 3 [RBF auditors] *find you here…!*” (DTC, CSCOM 1); “*We use to say “baara kèlaw”* 4 [the workers, in association with the auditors]*. They did not limit themselves to what we told them, they went to verify in the villages.*” (Communal health committee president, CSCOM 6). However, those elements of language are now part of the past since the “*buru futini*” and the “*RBF mogow/baara kèlaw*” discontinued at the end of the project.

A gap between the different roles (management vs. health care provision) was also illustrated in the language and seemed problematic: “*Us* [CSCOM’s personnel]*, it is technical and them* [ASACO and communes]*, they are community based, they have comprehensions of the community.*” (DTC, CSCOM 6); “*Every time they* [health practitioners] *talked with technical terms, if you were drowned, you had to make them stop and tell them: What do you want to say by that?*” (CC’s health commission member). This gap was limiting interactions between key stakeholders and obliged the CSREF to play an intermediary role in the communications between the CSCOM and the parity committee (PC)(formed by 10 members of both ASACO and communes), this weighted on the channels of communication: “*Us* [CSCOM personnel]*, we have to submit numbers, now the community* [communes and ASACO] *does not understand the numbers, now it is the manager’s team* [from the CSREF] *that is called by the mayors.*” (DTC, CSCOM 6). Despite a rapprochement during RBF, a gap still seemed to be present between the operational (health agents) and the community levels (ASACO and communes).

According to the different centres, many rituals were instituted or reinforced around activities due to RBF: planning meetings, hygiene practices, AS, etc. Nevertheless, the end of the project brought the lessening of those activities, which needed a certain level of investment. Many participants mentioned that cheaper rituals were still maintained: the use of minutes or administrative support, punctuality and better reception of the beneficiaries, the production of monthly reports or the attendance to monthly CSCOM meetings (some of these rituals being there already before RBF). The involvement of communes and CSREF diminished after the project, more especially concerning supervisions or meetings with the CSCOM. In a general point of view, there seemed to be insufficient time to integrate efficiently those rituals: “*The objective was to create habits for people to ensure that even after RBF… it seems to us that it ended before creating that habit.*” (CC’s health commission member).

#### Rules & procedures

“*Even when you cultivate your field, you must do a monitoring. As long as there is no monitoring, we harvest nothing.*” (Communal health committee president, CSCOM 6).

Many rules and procedures from RBF were integrated to the host organisation during the project’s implementation: habits linked to work quality (punctuality, kindness towards beneficiaries, use of registries, etc.) and some activities (SES, CPN or CPON). However, those components did not all continue after the project. According to different interviewees, the quality of the contact with beneficiaries and the use of registries were the elements that were the most sustained.

At the end of RBF, external supervision by the DRS and international NGO ceased while CSREF supervisions in CSCOM diminished (passing from 1/month to 1/year in different CSCOM). We can therefore presume a return to the old practices: “*It is the lack of internal follow up that brought us to fall back in the old practice.*” (Management member, CSREF 1). PCs and a health commissions (part of the CCs) were also reinforced during RBF with the objective to provide a better monitoring and involvement of elected representatives in the health sector. At the end of the project and more specifically at the end of the purchase of services, the participation of communes declined: “*We worked with this idea of competition, but until then we have slacked after, it is that mistake that got us.*” (Communal health committee president, CSCOM 6).

Almost the entirety of participants declared the RBF tasks were clear and corresponded to their prior tasks. With RBF, the tasks were nonetheless clarified which enabled the specialisation of some workers: e.g. the use of a specific community midwife for the CPN, the CPON or PF. Personnel engagement forms were signed linking workers to their tasks; yet, no collected data can demonstrate the sustainment of those forms. Notwithstanding those improvements, a decrease of CPON, SES and PF was noticed by stakeholders.

The inclusion of different RBF procedures’ manuals concerning the planning of the centres did not seem effective, since they mostly remained unutilised: “*If you go for an activity and that you have difficulties on the field… that we did not think about, then the y refer to me. But regarding the document… not really.*” (DTC, CSCOM 3). The project stayed disconnected to the health centres since it was considered as an external project with a definite duration and a strict focus on financial motivation: “*The objectives were not the same* [as before]*, because RBF, at that moment, you do activities and you gain something.*” (DTC, CSCOM 4). Despite the efforts made to unite actors within the health districts (CSCOM, ASACO, communes), every entity stayed independent regarding its planning, even if the health centres depended on these actors collaboration for their good functioning: “*…few micro-plans* [CSCOMs’ annual planning] *were integrated within communal budgets.*” (Management member, CSREF 2).

Being perceived as a winning formula, few modifications of procedures took place during the RBF project. Many initiatives came from health centres themselves with the aim to augment the purchase of their services: “*Even just between us, vaccinators, we held reunions. We talked about vaccination, good quality vaccination, vaccines maintenance… That was to increase our points.*” (Vaccinator, CSCOM 5); “*We required to all women in our health area to do the CPN… Those who do not respect those measures, we penalise you. Also, if you are coming for the vaccination of the child, we do not accept until you paid the penalty.*” (ASACO/treasurer, CSCOM test). According to different stakeholders, those modifications to RBF procedures show a certain pressure made by the purchase of services on health providers; even leading to data falsification (e.g. non provision of PF services, increased numbers of consultations, etc.).

#### Adaptation

“*In the first time, it was necessary to adapt people to get results.*” (CC’s health commission member).

In the context of individuals, much technical training was offered and matched the actual qualification needs of health providers: “*…the nurses, health agents that do not have a good level.*” (Management member, CSREF 1). Gains were perceived: “*When RBF arrived, I made trainings that enabled me to know how to fill in all those forms.*” (Pharmacy manager, CSCOM 3). However, those gains slowly disappeared with time and personnel rotation: “*Since RBF has stopped, there is more than a year, the ASACO did not call for one training… the old president* [of the ASACO] *has deceased and the new president did not received any training.*” (ASACO/treasurer, CSCOM test). It is worth noting that due to the political situation and the impossibility of the country to conduct local elections, much of the communal personnel (mayors and members of CC) was simply maintained, which helped sustain the expertise acquired by the local authorities during the project.

Some specific aspects of each health organisation were not taken into account during evaluations. This could then penalise certain types of centres. A centre with a smaller area of service could rapidly cap the possible quantity of services offered; a centre with a low population density would have higher costs for certain services (e.g. SA); or a centre with a large area of service could have difficulties in providing continued access to some quality services in remote villages or hamlets.

The project took into account the communities’ different needs while involving them in trimestral evaluations designed for the purchases of service. Elements such as quality of the greeting, the cost of prescriptions or the amount of time spent waiting were evaluated by the “*baara kèlaw*” (auditors) that went in different villages for the counter-evaluation. An evaluation of the needs in the Koulikoro region was also conducted (through another project) and enabled to “*… let* [to others the responsibility of] *nutrition, because we saw that nutrition, practically, was accounted for in our region.*” (Ex-DRS member). Already started with the creation of a communal essential information system (SIEC) (before RBF), the gathering of the stakeholders (CSCOM, ASACO, communes, CSREF, CC) around the health centres was facilitated, but faded at the end of the RBF project: “*It is shy now* [communes’ implication], *anyway… the collaborative relationship, anyway, stayed… the trust relationship*.” (DTC, CSCOM 2).

After being inspired by the implementation of RBF in Rwanda, the project was adapted to be more “*à la malienne*”: “*There is a mission that went in Rwanda for a study visit, but the political context being different… Therefore, we had to imagine all the tools necessary that could help to the implementation of the RBF project*.” (Management member, CSREF 2). Adaptation was mainly done to ensure the implication of the communities (communes and circles more especially) in the project since the Malian health system is highly decentralised. The project was therefore well adapted to the Malian health system general structure, using the hierarchy and organisations already in place for the RBF implementation.

At the operational level, the project was unanimously perceived as simple and compatible to the old tasks: “*It has nothing particular, it is our daily tasks that RBF wants us to improve.*” (Management member, CSREF 1). Tasks and procedures were all perceived as pertinent and with visible global effects (attendance to the centres, quality of services, investments, etc.). Almost no problem linked to the project was reported apart from its sudden ending which provoked a certain incomprehension and demotivation: “*The negative effect was the abrupt weaning… you know that with the end of RBF now, people have mostly giving up.*” (Management member, CSREF 1).

#### Development of relationships between stakeholders

“*They* [RBF team] *have developed within you the team work spirit, which has made our results better.*” (Health auxiliary, CSCOM 2).

RBF helped the gathering of the health centres’ stakeholders: health centres personnel and management committee, communities (communes and circles) and even other groups such as women, youth or religious associations and village chiefs. “*Those contacts exist between community health agents (CHA), those CHA, those rural maternities… but we had the link cut… with RBF we restored that, necessarily, since we had to unite to obtain results.*” (Medicine unit member, CSREF 1).

Communication channels opened during RBF following the hierarchy in the health system’s structure: “*When previsions are not achieved, the commune tells the ASACO to join its efforts to fill the gap. The ASACO also comes to tell the health agents to multiply their efforts to close the gap.*” (Community midwife, CSCOM 4). The communication was on the other hand centred on results to achieve. Communication during supervisions, evaluations and counter-evaluations were not always clear for health care providers: “*We have the impression that the guys* [CSREF auditors] *are against us. If we could have the same information that those guys. We have the impression that they are better equipped than us, that they have more information.*” (DTC, CSCOM 3). Those conflicts emerged because if the services did not meet quality criteria, they were not purchased and the personnel, as well as the health centre, could not benefit from the financial bonuses. Nevertheless, “*When there were errors, they* [CSREF auditors] *would show them to us* [CSCOM personnel]*, and we would correct them* [for the next purchase].” (Vaccinator 2, CSCOM 3) thus enabling a continued quality improvement process in the health centres.

While opening new communication channels, RBF also favoured team work. Even though some tasks became more specialised, a right to scrutiny was given to every actor and permitted a better adoption of the activities (from supervision to vaccination or deliveries) by the different actors (more precisely health agents): “*The change that it* [RBF project] *brought, it is to gather everyone… There are tasks that if only one person engage itself to do… it will not be done well. But when everyone* [CSCOM personnel] *works together, there is an exchange of experience and each one understands its mistakes and corrects itself.*” (Auxiliary, CSCOM 2). The presence of leadership was also linked to the notion of results and objectives to be attained.

“*If there is no more reunion, relationships, necessarily, it ends. There will be permutations… there will be rupture in the relations, necessarily.*” (CC’s health commission member). This situation post-RBF, concerns especially the supervision done by the CSREF and the DRS that was financed by the project. Communes and the CC also disengaged themselves at the end of the project, “*It* [the participation] *went down a bit, because the planning… we* [CSREF personnel] do not do it with them [CC] *anymore. In fact, they participate less to problems resolution now.*” (Hygiene unit member, CSREF 2); “*… if the ASACO has money, we* [the commune] *are easy* [there is less needs of implication in the CSCOM].” (Mayor, CSCOM 4). This could be caused by the end of the project’s funding and by a lack of motivation for the actors. During the project, both communes and ASACO did ask for individual bonuses, without success.

### Sustainability process

“*First we start by the diagnosis of the past activities, then identify the problems, do plans, gather the available resources to achieve the objectives, voilà!*” (Hygiene unit member, CSREF 2).

#### Evaluation of the integration capacity

An evaluation of capacities was made prior to the implementation of the project in each of the participating centres. This evaluation was conducted with the participation of the different project’s stakeholders. The lack of material and human resources were identified at all levels of the health system: qualified personnel and a variety of material (medical, logistical, etc.) in the health centres, vehicles for supervisions (DRS and CSREF), human and financial resources (DNS). “*The division that took care of RBF* [the DNS’s Sanitary Establishments division] *was not very developed* [in terms of personnel and financing].” (Ex-DNS member). Apart from the Dutch development aid funds spent for the project, few resources are given today to stabilise the project’s achievements. This lack of resources was also reflected through leadership that was not shared between local authorities and the project management in regards to the capacities evaluation (and throughout the project): “*The government does not have the means, this is why since that thing* [RBF project] *exists, it was not involved*.” (ASACO/treasurer, CSCOM 3). The abandonment of the baseline study illustrates both the lack of resources and leadership of the Malian government. In fact, after the 2012 *coup d’État*, USAID (United-States agency for international development) withdrew its funds for the baseline study, of which the State did not take responsibility “*…at this moment, there was no money in the system* [for the baseline study]*… there was no resource.*” (Ex-DRS member).

To better understand the needs of the stakeholders, the project used a diversity of evaluations: “*There are observations… demographic and health queries that are made every 5 years, therefore tendencies. There are also partners that conduct studies.*” (Ex-DRS member). More locally, an evaluation of the health centres was conducted for the first results’ planning workshop, “…*they* [SNV] *told us* [CSCOM personnel] *to gather the 3 months* [reports] *prior RBF to see at what level we were.*” (DTC, CSCOM 6). The project aligned itself on population needs assessments that could be vague, as well as service evaluations that could be unrepresentative with tendencies of not taking into account monthly or seasonal specificities: “*During dry season, CSCOMs have a lower performance* [attendance].” (ASACO/president, CSCOM 1). Thanks to earlier projects with SNV in the region such as the creation of a SIEC (without links with the subsequent RBF project), the evaluation of the relationships between actors (ASACO, communes, CSCOM) was already made and actions had already been taken to resolve some difficulties: lack of communication between actors, lack of confidence in the budget management and lack of involvement in the CSCOM (by the ASACO or communes). A design mapping was also made by the KIT to position itself and better implement the project.

#### Sustainability planning

Little attention was given to planning sustainability since the project was considered as experimental and therefore with a definite duration; the idea of scaling up was however further developed according to different documents from the project [35–37]. This is explained by the fact the project took place in parallel to rumours of a potential World Bank (WB) funding for the scaling up of the project in the Koulikoro region^5^ [40]. The designation of the project even alternated from pré-pilote to pilot: “*The idea was that with the bank* [WB], *there will be a greater RBF project and we* [DNS] *did not wish things to be parachuted like that.*” (Ex-DNS member); “*The WB will always say that it will do a pilot, therefore we said, since the bank will do a pilot project, we will say that we have done a pré-pilote. It is just positioning yourself…*” (Ex-member, SNV) aiming to ensure a RBF “*à la malienne”*.

The implementation and scaling up’s planning did not correspond. Shortcuts were used during the pilot project with the participation of external organisations having the role of counter-verifying results (independent NGOs) and managing the purchase of services (SNV/KIT): “*In the reflexions that we* [SNV/KIT] ma*de for our pilot… it was to transform the “Agence Nationale d’Évaluation des Hôpitaux” into a national agency for RBF… for verification, instead of creating other structures*.”^6^ (Ex-SNV member); “*Regarding the question of sustainability, are we continuing like that? We must see for the normal structures of financing in Mali.*” (Ex-DNS member).

#### Execution of action towards sustainability

Few actions were taken to sustain the RBF project, this was due to a lack of planning and appropriation by some actors: “*The final evaluation* [of the project] *interrogated all structures* [national level: DNS and MHPH]*, people are very positive, but you do not feel that in their manners, their actions.*” (Ex-SNV member). The high degree of personnel and project’s leaders’ rotation did not facilitate action towards sustainability. Still some exceptions remain: “*Yesterday* [February 4^th^, 2016]*, I called out the community health agents to ensure data did not fall, because if data would decrease, it will impact on the money* [the CSCOM revenue]*… We planned meetings in villages and hamlets also, to ensure data are not falling.*” (Mayor, CSCOM 4).

#### Evaluation of sustainability and modifications

It was rumoured that the WB might scale up this RBF project by expanding it to other sites, however there was little evidence that sustainability and the future of the original pilot project was being considered: “*I think for the budget it had a beginning and an end… So I think if you look purely at financial sustainability, it was not financially sustainable. But taking into account the political developments and that the World Bank was planning to roll out a national system, albeit with a different FBR framework that KIT had issued, there would have been an opportunity to continue the FBR program in Koulikoro.*” (Member 2, KIT).^7^ Sustainability’s determinants and its process illustrate few events inclined to facilitate the project’s sustainability, and this was evident in the different implementation contexts of the project. This supports therefore the final result saying that this RBF project has a weak degree of sustainability.

## Discussion

This study illustrates that many gains were generated through the implementation of the RBF project: long term investments in human and material resources (organisational memory), correspondence between objectives (values/codes), integration of different tasks and procedures (rules/procedures), capacity to adapt to different contexts (adaptation) and creation of a trust relationship between actors (development of relationships between stakeholders). However, other components of the project show that sustainability remains at a weak level, notably through the insufficiency of stable resources, a lack of supervision and a loss of contact between the stakeholders. In regards to the process of sustainability, planning was centred on a potential scaling up by an external actor (WB) rather than on the sustainability of the actual project.

Results represent the importance of adapting the project to all the different implementation contexts to be able to sustain it: i) intervention (e.g. simple functioning), ii) individuals (e.g. to grant benefits to all actors involved in the implementation), iii) organisation (e.g. the investments in the health centres), iv) local environment (e.g. to take into account populations’ needs like the cost of prescriptions), v) external system (e.g. the utilisation of the health system’s structure already in place). Since all those contexts are vital for the implementation of the project, they must be taken into account in a systematic way so as to sustain the project [23, 41, 42]. While acknowledging the importance of the diversity of stakeholders from the different implementation contexts, this research choose to focus on more local contexts (intervention, individuals, and organisation) and to explore to a lesser extent the local environment or external system [See Table 2]. This was mainly due to the more difficult access to participants related to those contexts. Therefore, other studies need to look at those implementation contexts (ex.: the agenda-setting at the different political levels) in regards to sustainability and to go beyond the organisational or individual contexts that are more often studied [23].

The end of the project was a major event for this pilot, after which many activities (or routines) ceased or rapidly diminished (supervisions, meetings, SA, SES, etc. depending of the health centre). Sustained routines such as the use of certain registries, the punctual opening of services, or the kindness towards the beneficiaries were perceived with very low cost and often emerged from the organisations (or from specific individuals) as solutions to obtain better results for the project. On the contrary, the discontinued routines were often referred as costly (ex.: high maintenance costs to provide SA in more remote villages) and were new activities in the organisation, even if they were part of the PMA. While the cost of maintaining or not a routine seemed an important determinant of sustainability, some other low cost routines were also discontinued (ex.: internal and external meeting, supervisions). Going beyond the simple financial explanation, the issue of motivation then arises to better comprehend why some low cost routines were sustained and some not.

The motivation (someone’s mobilisation to act [43]) to sustain or not a new routine can be analysed through the theory of self-determination [44] where different categories are created in relation to the perceived locus of causality of the motivation, the autonomy of individuals (to be a causal agent of one’s life) and other components of motivation. According to this theory, types of motivation range on a continuum from nonself-determined motivation with more external locus of causality and more controlled motivation (ex.: based on external rewards such as primes, trainings, supervisions, someone’s ego, etc.); to self-determined motivation with more internal locus of causality and more autonomous motivation (ex.: based on the pleasure to conduct an activity or the correspondence between someone’s behaviour and identities, beliefs or values) [45–47]. These types of motivation thus respond to three main needs someone has: relatedness (the desire to interact/connected to others), autonomy (the ability to be a causal agent of one’s life) and competence (the sentiment of efficacy in someone’s interaction with its environment) [44, 47].

While acknowledging the fact that every individual adopt different sources of motivation, we can see that the perceived locus of causality were mainly external and linked to results rather than the activities themselves: individual bonuses, supervision by “*RBF mogow*”, trainings and feedbacks from supervisions, competition between CSCOM and long/short term investments in the work place [45, 47–51]. For communes, perceived sources of motivation were more linked to someone’s reputation (in regards to the next election) and the desire to make a difference in the community. However, while the project aimed at involving communes in the management of the health centres, few decisions could be made by these actors (due to the lack of knowledge or the language gap explained earlier). These types of motivations represent a low level of autonomy since they are mainly based on external source of motivation, which are predetermined by the project’s design and processes and stopped at the end of the project. However, working in a poorly funded health system, those actors can easily recognize the inadequacy of their salary and still appreciate the increase of their income through bonuses [48–52].

By looking at the more sustained activities (new registries, punctuality, better reception), some can see that more intrinsic sources of motivation are also present such as the enjoyment of challenges and tasks. For examples, new registries were created in a maternity during the project as a solution to the problem of undocumented results and were sustained with the help of a “champion” [53–55]; by clearly dividing the tasks of the SA (CPON, CPN, vaccination, SES…) and by being more equipped for different tasks (through trainings, materials, infrastructures), health workers seemed to better appreciate doing new routines and helped sustain them after the end of the project (SA, vaccination campaigns, punctuality at work, better reception).

While different types of motivation were present through the RBF pilot project, new routines with a more autonomously driven motivation seem more sustainable. A major reason is the instability (or lack of control) of a source of motivation for health workers (ex.: delayed payments [56] or the “*brutal*” stop of the project’s funding), which is often dependant on international organisations’ funding that habitually vanishes at the end of the project [28, 42]; this being the case in Mali, in Benin and Burkina Faso [17, 26, 56, 57]. Without this financial source of motivation (ex.: the bonuses and organisational investments), activities are discontinued, which therefore brings a lack of sustainability for the project. In a context of optimisation of limited resources in global health (ex.: time, human or financial resources), actors’ motivation should be more autonomously driven since it can be less reliant on instable resources and can push towards activities’ ownership and sustainability [46, 47].

A way to provide such autonomous motivation could be to focus more on the different actors’ appropriation of the new routines rather than the results. Like other RBF project [38, 58, 59], the pilot in Mali aimed to increase the participation of the different community stakeholders in the health centres’ management; which could correspond to someone’s need of relatedness. However, the roles attributed to some stakeholders (especially the communes) for the project’s activities were not sufficient to ensure their interest (intrinsic motivation) in the long term. While pushing for more autonomy of health centres, the role of international organisations can be too great and hinder local actors’ appropriation of the project, as it is illustrated by the budget management of the RBF project by the KIT, rather than the relevant national authorities (communes and CC). The project can easily be thought to be under the responsibility of the external funders, who can be tempted to order decisions concerning the project, and therefore diminish the role of local actors in the decision-making process [41]. The type of services bought, the global targets to achieve, the way of monitoring, reporting or evaluating are all different examples of elements being reported as non-negotiable in RBF projects in South-Africa, Tanzania or Zambia [5]. Other projects in the health sector also illustrate this project approach where the intervention was often said to be “*forced*” or “*superimposed*” in the host organisation [17, 20, 26, 42, 56]. Without the ability of adapting the project to the organisation, individuals can be forced to live incongruities between their goals, identities or values and their roles in the new routines [47]; therefore, routines become harder to sustain.

These results can partly be transferred to different projects in Africa and more specifically to West Africa, since there are many organizations (African Union, ECOWAS) for the convergence of the region with regards to public policies [60]. However, it is important to note that Mali is a highly decentralised country in regards to its health care services organisation, which is not always the case in other contexts. Many interventions have also been put in place prior and in parallel to the RBF project (e.g. creation of the SIEC, investments made by other projects/organisations) creating a favourable environment for its implementation through stakeholders cooperation. A scale up strategy for RBF outside the region of Koulikoro, where such interventions have not been conducted, could lead to an overestimation of the effects of its scale up.

A first limit to this study is the memory bias of the participants which could not always recall all the specific facts due to the gap between the end of the project and the data collection period. A greater number of participants were then recruited to limit this bias and to triangulate data. The collect of critical events also reduces that bias since those events were more easily identifiable and were collectively shared by the actors. A second limit could be the perceived role of the main researcher (MS) by the participants. Despite the clarification of the researcher’s role before every interview, many participants would still associate him to a project evaluator, which was apparent in the data (quasi-absence of negative information concerning the project and reluctance to share that information). Since the participants had often directly or indirectly benefited from the project, they often were in conflict of interest; they still had hope of the project’s return (mainly monetarily). Collected data can therefore be influenced by the financial relationship participants had with the RBF project, as it has been seen elsewhere [61]. The sustainment of activities, relationships or acquisitions and integrated resources could then be overestimated, leading to an even lower level of sustainability.

## Conclusion

If better understood, the process of sustainability could help answer many actual needs linked to the project implementation (e.g. results optimisation, better usage of resources in the long run). Like other projects [20, 26], the RBF project in Mali is characterised with a weak level of sustainability. While many efforts were made to adapt the project to the national health systems and while much investment was made in the different organisations, the absences of some sustainability determinants as well as the failure of certain phases of the sustainability process do provide different ways of explaining the lack of sustainability of this project. A better planning of sustainability would enable projects to better maintain their achievements and to ensure continued motivation, better integration of tasks and appropriation by stakeholders. This study illustrates the need to deepen our understanding of the concept of sustainability to obtain a better definition of its determinants and continued phases of sustainability.

## Abbreviations

AS: Advanced strategies *(Stratégies Avancées)*
ASACO: Community health associations *(Assemblée de santé communautaire)*
CC: Cercles’ councils *(Conseil du Cercle)*
CPN: Prenatal care *(Consultation prénatale)*
CPON: Postnatal care *(Consultation postnatale)*
CSCOM: Community health centres *(Centre de santé communautaire)*
CSREF: Referral health centres *(Centre de santé de reference)*
DNS: National direction for health *(Direction nationale de la santé)*
DRS: Regional direction for health *(Direction régionale de la santé)*
DTC: CSCOM’s technical director *(Directeur technique du CSCOM)*
ECOWAS: Economic Community of West African States
FP: Family planning
KIT: Royal Tropical Institute *(Koninklijk Instituut voor de Tropen)*
MHPH: Ministry of Health and Public Hygiene
NÉPAD: New Partnership for Africa’s Development
NGO: Non-governmental organisations
PC: Parity committee *(Comité paritaire)*
PMA: Minimum package of activities
RBF: Results-based financing
SES: Healthy children’s monitoring *(Suivi des enfants sains)*
SIEC: Communal essential information system*(Système d’information essentielle communal)*
SNV: Dutch Cooperation *(Stichting Nederlandse Vrijwilligers)*
WB: World Bank

## Footnotes

1
*Italic* quotes have been freely translated from French (or Bambara) by the main researcher (or the local translator) who did the data collection.

2 Bambara vocabulary

3 Idem

4 Idem

5 Rumours have been confirmed with the WB implementing another pilot project for the whole region of Koulikoro. This pilot took place for only 3 months (October to December 2016) due to different delays. There is no sign in this last report if a RBF scale-up will be implemented.

6 The Agence National d’Évaluation des Hôpitaux did not serve during RBF, it was instead independent NGOs that had the role of being external auditors.

7 At the moment of the writing of this article, no information demonstrates that the WB will start another RBF project in Mali.

## References

[1] Soeters R, Habineza C, Peerenboom PB (2006). Performance-based financing and changing the district health system: experience from Rwanda. Bull World Health Organ.

[2] Chimhutu V, Tjomsland M, Songstad NG, Mrisho M, Moland KM (2015). Introducing payment for performance in the health sector of Tanzania- the policy process. Glob Health.

[3] Bonfrer I, Soeters R, Van de Poel E, Basenya O, Longin G, van de Looij F, van Doorslaer E (2014). Introduction of performance-based financing in burundi was associated with improvements in care and quality. Health Aff.

[4] Mokdad AH, Colson KE, Zúñiga-Brenes P, Ríos-Zertuche D, Palmisano EB, Alfaro-Porras E, Anderson BW, Borgo M, Desai S, Gagnier MC (2015). Salud Mesoamérica 2015 initiative: design, implementation, and baseline findings. Popul Health Metrics.

[5] Barnes A, Brown G, Harman S (2015). Locating health diplomacy through African negotiations on performance-based funding in global health. J Health Diplomacy.

[6] Beane CR, Hobbs SH, Thirumurthy H (2013). Exploring the potential for using results-based financing to address non-communicable diseases in low-and middle-income countries. BMC Public Health.

[7] Ireland M, Paul E, Dujardin B (2011). Can performance-based financing be used to reform health systems in developing countries?. Bull World Health Organ.

[8] Paul E, Sossouhounto N, Eclou DS (2014). Local stakeholders' perceptions about the introduction of performance-based financing in Benin: a case study in two health districts. Int J Health Policy Manag.

[9] Turcotte-Tremblay A-M, Gautier L, Bodson O, Sambieni NkE, Ridde V: Dans les coulisses du pouvoir décisionnel : le rôle des organisations internationales dans l'expansion du financement basé sur les résultats dans les pays à faible et à moyen revenu. 2016.

[10] Fritsche GB, Soeters R, Meessen B. Performance-based financing toolkit: World Bank Publications; 2014.

[11] Eichler R: Can “pay for performance” increase utilization by the poor and improve the quality of health services. Background papers for the Working Group on Performance Based Incentives 2006.

[12] Basinga P, Mayaka S, Condo J (2011). Performance-based financing: the need for more research. Bull World Health Organ.

[13] Das A, Gopalan SS, Chandramohan D (2016). Effect of pay for performance to improve quality of maternal and child care in low- and middle-income countries: a systematic review. BMC Public Health.

[14] Oxman AD, Fretheim A (2008). An overview of research on the effects of results-based financing.

[15] Turcotte-Tremblay A-M, Spagnolo J, De Allegri M, Ridde V (2016). Does performance-based financing increase value for money in low- and middle- income countries? A systematic review. Heal Econ Rev.

[16] Basinga P, Gertler PJ, Binagwaho A, Soucat ALB, Sturdy J, Vermeersch CMJ (2011). Effect on maternal and child health services in Rwanda of payment to primary health-care providers for performance: an impact evaluation. Lancet.

[17] Paul E: Marché de services relatifs à la réalisation d'une étude sur la viabilité et la pérennisation de l'approche du financement basé sur les résultats (FBR) au Bénin. In*.*: CTB et Agence belge de développement; 2016.

[18] Scheirer MA, Dearing JW (2011). An agenda for research on the sustainability of public health programs. Am J Public Health.

[19] St Leger L (2005). Questioning sustainability in health promotion projects and programs. Health Promot Int.

[20] Ridde V, Pluye P, Queuille L (2006). Évaluer la pérennité des programmes de santé publique : un outil et son application en Haïti. Rev Epidemiol Sante Publique.

[21] Pluye P, Potvin L, Denis J-L (2004). Making public health programs last: conceptualizing sustainability. Eval Program Plan.

[22] Johnson K, Hays C, Center H, Daley C (2004). Building capacity and sustainable prevention innovations: a sustainability planning model. Eval Program Plan.

[23] Moullin J, Sabater-Hernandez D, Fernandez-Llimos F, Benrimoj S (2015). A systematic review of implementation frameworks of innovations in healthcare and resulting generic implementation framework. Health Res Policy Syst.

[24] Chambers D, Glasgow R, Stange K (2013). The dynamic sustainability framework: addressing the paradox of sustainment amid ongoing change. Implement Sci.

[25] Pluye P. Vers un nouveau modèle théorique du déroulement des programmes : étude de la routinisation des programmes en promotion de la santé: Université de Montréal; 2002.

[26] Mallé Samb Oumar RV, Ludovic Q (2013). Quelle pérennité pour les interventions pilotes de gratuité des soins au Burkina Faso ?. Revue Tiers Monde.

[27] Pluye P, Potvin L, Denis J-L (2000). La pérennisation organisationnelle des projets pilotes en promotion de la santé. Ruptures, Revue Transdisciplinaire en Santé.

[28] Olivier de Sardan J-P, Diarra A, Koné FY, Yaogo M, Zerbo R (2015). Local sustainability and scaling up for user fee exemptions: medical NGOs vis-à-vis health systems. BMC Health Serv Res.

[29] Khim K, Ir P, Annear PL (2017). Factors driving changes in the design, implementation, and scaling-up of the contracting of health Services in Rural Cambodia, 1997–2015. Health Syst Reform.

[30] Sieleunou I, Turcotte-Tremblay A-M, Yumo HA, Kouokam E, Fotso J-CT, Tamga DM, Ridde V (2017). Transferring the purchasing role from international to National Organizations during the scale-up phase of performance-based financing in Cameroon. Health Syst Reform.

[31] Shroff ZC, Tran N, Meessen B, Bigdeli M, Ghaffar A (2017). Taking results-based financing from scheme to system. Health Systems & Reform.

[32] Yin RK (2013). Case study research: design and methods: sage publications.

[33] Etat de santé et tendances. http://www.aho.afro.who.int/profiles_information/index.php/Mali:Health_Status_and_Trends/fr. Accessed Oct 26 2017.

[34] Touré L (2011). La gouvernance de la santé dans la région de Sikasso. Bamako: MISELI.

[35] Toonen J, Dao D, Matthijssen J, Koné B: Évaluation finale: Accélérer l'atteinte de l'OMD 5 dans la région de Koulikoro - Projet pilote financement basé sur les résultats dans les cercles de Dioïla et Banamba. In*.*: Institut Royal Tropical; 2014.

[36] Dao D, Toonen J, Koné B: Contribution du FBR à la bonne gouvernance des centres de santé communautaire au Mali. In*.*; 2014.

[37] Toonen J, Kone B, Dao D: Le Financement Basé sur les Résultats (FBR) au Mali. In*.*: KIT; s.d.

[38] Renmans D, Holvoet N, Criel B, Meessen B (2017). Performance-based financing: the same is different. Health Policy Plan.

[39] Étude de la pérennisation des résultats du projet pilote de FBR dans les districts sanitaires de Fana et Dioïla. http://www.equitesante.org/financement-base-sur-les-resultats-en-sante-maternelle-et-infantile-et-equite-au-mali-et-au-burkina-faso/. Accessed Oct 26 2017.

[40] The World Bank: Implementation completion and results report (IDA - H7530) on a grant in the amount of SDR 19 million (US$ 30 million equivalent) to the Républic of Mali for a strengthening reproductive health project (SRHP). In*.* s.l.: World Bank; 2017: 81 pages.

[41] de Renzio P, Whitfield L, Bergamaschi I (2008). Reforming foreign aid practices: what country ownership is and what donors can do to support it.

[42] Castellanet C (2003). Cycle des projets, cadre logique et efficacité des interventions de développement.

[43] Ryan RM, Deci EL (2000). Self-determination theory and the facilitation of intrinsic motivation, social development, and well-being. Am Psychol.

[44] Deci EL, Ryan RM (2008). Self-determination theory: a macrotheory of human motivation, development, and health. Can Psychol.

[45] Forest J, Mageau GA (2008). La motivation au travail selon la théorie de l’autodétermination. Psychologie Québec.

[46] Forest J: Comment rendre un chercheur heureux, performant et pour longtemps. In: Dossier Santé psychologique des chercheurs*.* Edited by Découvrir: Association francophone pour le savoir-Acfas; 2016.

[47] Lohmann J, Houlfort N, De Allegri M (2016). Crowding out or no crowding out? A self-determination theory approach to health worker motivation in performance-based financing. Soc Sci Med.

[48] Bertone MP, Lagarde M, Witter S (2016). Performance-based financing in the context of the complex remuneration of health workers: findings from a mixed-method study in rural Sierra Leone. BMC Health Serv Res.

[49] Khim K. Are health workers motivated by income? Job motivation of Cambodian primary health workers implementing performance-based financing. Global Health Action. 2016;9. doi:10.3402/gha.v9.31068. 10.3402/gha.v9.31068 PMC4913167 27319575

[50] Meessen B, Kashala J-PI, Musango L: Output-based payment to boost staff productivity in public health centres: contracting in Kabutare district, Rwanda. Bull World Health Organ 2007, 85(2):108-115. 10.2471/BLT.06.032110 PMC2636284 17308731

[51] Ensor T, Chapman G, Barro M: Paying and motivating CSPS staff in Burkina Faso: evidence from two districts. Initiative for Maternal Mortality Programme Assessment Aberdeen, Scotland: University of Aberdeen 2006.

[52] Olivier de Sardan J-P (2014). La routine des comportements non-observants au sein des services publics Nigériens. Connaître la culture bureaucratique pour la réformer de l'intérieur.

[53] Shediac-Rizkallah MC, Bone LR (1998). Planning for the sustainability of community-based health programs: conceptual frameworks and future directions for research, practice and policy. Health Educ Res.

[54] Damschroder LJ, Aron DC, Keith RE, Kirsh SR, Alexander JA, Lowery JC (2009). Fostering implementation of health services research findings into practice: a consolidated framework for advancing implementation science. Implement Sci.

[55] Cresswell K, Sheikh A (2013). Organizational issues in the implementation and adoption of health information technology innovations: an interpretative review. Int J Med Inform.

[56] Ridde V, Yaogo M, Zongo S, Somé P-A, Turcotte‐Tremblay A‐M. Twelve months of implementation of health care performance‐based financing in Burkina Faso: A qualitative multiple case study. Int J Health Plann Mgmt. 2017. https://doi.org/10.1002/hpm.2439. 10.1002/hpm.2439 PMC5900741 28671285

[57] Paul E, Lamine Dramé M, Kashala J-P, Ekambi Ndema A, Kounnou M, Aïssan JC, Gyselinck K. Performance-Based Financing to Strengthen the Health System in Benin: Challenging the Mainstream Approach. Int J Health Policy Manag. 2017(6):1–13. 10.15171/ijhpm.2017.42 PMC5745866 29325401

[58] Falisse JB, Meessen B, Ndayishimiye J, Bossuyt M (2012). Community participation and voice mechanisms under performance-based financing schemes in Burundi. Tropical Med Int Health.

[59] Falisse J-B, Vergeer P, Gebre Medhin JKG, Juquois M, Akpamoli A, Robyn J, Shu W, Zabiti M, Hassan R, Jallow B et al: Community results-based financing in health practice : reflections on implementation from experiences in six countries. In: The Health, Nutrition and Population Global Practice Knowledge Briefs of the World Bank. Online: World Bank Group; 2017: 4 pages.

[60] Olivier de Sardan J-P, Ridde V (2015). Diagnosis of a public policy: an introduction to user fee exemptions for healthcare in the Sahel. BMC Health Serv Res.

[61] Turcotte-Tremblay A-M, Spagnolo J, De Allegri M, Ridde V (2015). Does performance-based financing increase value for money in low-and middle-income countries? A systematic review. Heal Econ Rev.

